# Workplace ostracism and political behavior: a moderated mediation model of moral disengagement and supervisor-rated job performance

**DOI:** 10.3389/fpsyg.2026.1838746

**Published:** 2026-05-21

**Authors:** Xuan Yu, Maoting Yang, Hantai Zhang

**Affiliations:** 1 School of Economics and Management, Southwest Petroleum University, Chengdu, China; 2 Antai College of Economics and Management, Shanghai Jiao Tong University, Shanghai, China

**Keywords:** moral disengagement, political behavior, social cognitive theory, supervisor-rated job performance, workplace ostracism

## Abstract

**Introduction:**

Workplace ostracism is common in many organizations and has grown to be a significant factor affecting employees' attitudes and behaviors. This study investigates the mediating role of moral disengagement and the moderating role of supervisor-rated job performance in the relationship between workplace ostracism and employees' political behavior.

**Methods:**

Questionnaires were used to collect data from 408 Chinese employees across various industries. MPLUS and SPSS were used to test the model fit and hypotheses, respectively.

**Results:**

The results showed that workplace ostracism positively affects political behavior through moral disengagement. Additionally, the effect of workplace ostracism on moral disengagement and the indirect effect of moral disengagement are both negatively moderated by supervisor-rated job performance.

**Discussion:**

In addition to offering theoretical insights into the mechanisms underlying workplace ostracism and employees' political behavior, this study has practical implications for organizations looking to control organizational political behavior by improving performance evaluation systems and addressing employees' moral disengagement.

## 1 Introduction

Conflicts of interest and interpersonal friction within the organization are unavoidable, and workplace ostracism is growing more frequent as organizational structures diversify more quickly and workplace competition increases (Ali et al., 2020; Howard et al., 2020). In the United States, nearly 70% of employees reported having experienced some degree of exclusion during their career (O'Reilly et al., 2014). Similarly, industry surveys conducted by Zhaopin, a leading Chinese recruitment platform, suggest a comparable prevalence among employees in China. This phenomenon is common across many organizations and has many detrimental effects on individuals, teams, and organizations. Numerous studies have shown that workplace ostracism reduces job engagement (Xu et al., 2020), job performance (De Clercq et al., 2018; Feng et al., 2019), and creativity (Kwan et al., 2018; Tu et al., 2019). It may even lead to psychological problems such as anger (Bilal et al., 2020), anxiety (Ferris et al., 2008; Samma et al., 2020), and emotional exhaustion (Choi, 2019; Thompson et al., 2020). Individuals frequently use a variety of coping strategies, such as pro-job unethical behavior (Liu et al., 2025), pro-organizational unethical behavior (Zhang, 2020), counterproductive behavior (Ali Awad and Mohamed El Sayed, 2023; Zhu and Zhang, 2021), social cyberloafing (Hu et al., 2023), and knowledge hiding behavior (Chaudhary et al., 2024; Riaz et al., 2019), when faced with the suffering and pressure resulting from workplace ostracism. However, individuals' strategic behaviors within organizational power structures have received less attention in these studies, which have mostly concentrated on ethical or performance-related domains.

Employees' political behavior is a common coping action in organizational settings (Ferris and Kacmar, 1992), defined as the use of tactical influence by individuals that is rational, conscious, goal-oriented, and focused on furthering one's own interests (Valle and Perrewe, 2000). From this definition, political behavior is distinguishable from the aforementioned coping behaviors by its simultaneous integration of three core characteristics: strategic nature, goal orientation, and self-interest focus. In prior research examining coping responses to workplace ostracism, scholars have typically emphasized only one of these dimensions rather than exploring the synergistic integration of all three. As a multi-dimensional construct, political behavior is likely to serve as a more covert and prevalent coping mechanism for individuals confronting workplace ostracism, thereby offering a more systematic lens through which to understand how employees navigate exclusion in organizational settings. Therefore, this study further investigates the effects of workplace ostracism, concentrating on an additional outcome at the individual behavioral level: employees' political behavior. Several studies have shown that employees will use a variety of strategies to safeguard their own interests in unfavorable work environments (Qi et al., 2020; Prince et al., 2023; Yuan et al., 2022). Therefore, when faced with workplace ostracism in a highly competitive work environment, employees may turn to politically charged behaviors that safeguard their own interests in an effort to reclaim recognition and elevate their professional status. Political behavior is what these behaviors are. Classic studies on organizational politics have indicated that political behavior can be either functional or dysfunctional, depending on intent, tactics, and context (Mintzberg, 1983; Ferris et al., 2002). This study mainly focuses on the dysfunctional and self-interested aspects of political behavior. Such political behavior may lead to interpersonal deviance (Barlett et al., 2016), job burnout (Cropanzano et al., 1997), emotional exhaustion, and decreased employees' job performance (Hochwarter et al., 2007). Therefore, in order to implement effective management strategies, organizational managers must comprehend whether, how, and when workplace ostracism triggers employees' political behavior. Nevertheless, this particular phenomenon has not been studied in previous research.

According to social cognitive theory, the relationship between the external environment, individual cognition, and individual behavior is interactively determined (Bandura, 1986). Workplace ostracism is a common environmental factor that can affect an individual's behavior by triggering their cognitive state (Kuo and Wu, 2022; Liu et al., 2025; Spoor and Williams, 2007). As a cognitive tendency in individuals, moral disengagement may be a key mediating factor in this process. Moral disengagement is the process of cognitive restructuring that enables individuals to detach themselves from their moral standards and behave unethically without experiencing pain (Bandura, 1999). It is worth noting that the moral disengagement mechanism exhibits unique adaptability in explaining political behavior. Extant research has demonstrated that moral disengagement can facilitate pro-group unethical behavior (Zhang et al., 2023) and pro-job unethical behavior (Liu et al., 2025). However, political behavior differs in motivation. Pro-group unethical behavior is geared toward advancing organizational interests, while pro-job unethical behavior focuses on promoting professional interests. In contrast, political behavior is explicitly centered on promoting one's own interests, such as influence, resources, and power. More importantly, political behavior inherently entails strategic thinking, planning, and tactical implementation, and moral disengagement plays a proactive and pivotal role in this process. Specifically, it enables individuals to pre-justify self-interested actions as strategically necessary and normatively acceptable, thereby alleviating potential moral conflicts prior to behavior implementation. Consequently, moral disengagement is particularly well-suited for understanding political behavior, which is characterized by its strategic nature and self-interest orientation. Building on this theoretical logic, this study aims to explore the internal mechanism through which workplace ostracism influences employees' political behavior via moral disengagement.

Furthermore, we argue that supervisor-rated job performance, which is a crucial organizational management tool, serves as a significant contextual factor influencing individual behavioral decisions in the situation of workplace ostracism as a stressor. We suggest that supervisor-rated job performance, as a latent critical resource, may change employees' perceptions of workplace ostracism and their coping strategies, drawing on conservation of resources theory (Hobfoll, 1989). In the end, it has a significant moderating effect on the process by which employees' political behavior is triggered by workplace ostracism.

Based on the above empirical findings and theoretical perspectives, this study aims to investigate the effect of workplace ostracism on employees' political behavior, the mediating role of moral disengagement, and the moderating effect of supervisor-rated job performance. The main contributions of this study are as follows: First, it broadens the scope of workplace ostracism's outcome dimension. Employees who are experiencing workplace ostracism can improve their circumstances by engaging in political behavior, providing a fresh interpretation of the logic of such political behavior for excluded employees. Second, we identified the mechanism that connects workplace ostracism and political behavior, highlighting the mediating role of moral disengagement. This role is consistent with social cognitive theory's theoretical framework (Bandura, 1986), which aids in clarifying the fundamental reasoning behind how workplace ostracism affects political behavior. Finally, previous studies have predominantly examined the relationship between workplace ostracism and employees' job performance by treating the latter as an outcome variable (Imran et al., 2023; Kuo and Wu, 2022; Wang and Lai, 2023). However, in order to investigate its effect on the process by which workplace ostracism affects employees' political behavior through moral disengagement, this study used supervisor-rated job performance as a moderator. By doing this, this study contributes significantly to the existing literature regarding supervisor-rated job performance as a moderator.

## 2 Theoretical background and hypotheses

### 2.1 Workplace ostracism and political behavior

Workplace ostracism refers to the feelings of being excluded, ignored, isolated, or rejected by others in the workplace (Ferris et al., 2008). Research indicated that workplace ostracism, as a stressor, leads to losses in resources such as wellbeing (Jang and Chen, 2022), vitality (Rabiul, 2024; Wang et al., 2023), and creativity (Kwan et al., 2018; Tu et al., 2019) of the excluded individuals. In uncertain and ambiguous environments, political behavior is considered an important strategy for employees to obtain critical resources (Sun and Chen, 2017; Valle and Perrewe, 2000). Employees can successfully acquire a variety of instrumental resources through political behavior, such as material rewards, status, recognition, favor, and reputation (Cheng et al., 2022; Drory and Romm, 1990; DuBrin, 2009; Harris et al., 2007; Landells and Albrecht, 2017). Employees can gain leadership-level resources or support by using political behavior as a social skill, which strengthens their competitive advantage within the organization (Lin et al., 2022). Therefore, employees who are experiencing workplace ostracism are more likely to engage in political behavior to regain support and resources.

According to social cognitive theory, the relationship between the external environment, individual cognition, and individual behavior is interactively determined (Bandura, 1986). Employees who feel excluded at work frequently experience changes in their internal cognition and emotions, such as a diminished sense of belonging (Brison and Caesens, 2023) and heightened job insecurity (Zhang et al., 2024). Employees frequently reassess their standing within the organization and their access to resources to safeguard their interests, believing that engaging in political behavior will help them. They see political behavior as a successful adaptive strategy. Additionally, research has shown that employees often adopt these behaviors as a way to protect themselves in unfavorable work environments (Ming et al., 2022; Valle and Perrewe, 2000).

Based on these arguments, this study proposes the following hypothesis:

Hypothesis 1 (H1). Workplace ostracism has a positive effect on political behavior.

### 2.2 The mediating role of moral disengagement

The concept of moral disengagement originates from Bandura's (1986) social cognitive theory, which posits that moral disengagement is a psychological mechanism through which individuals regulate the relationship between their behavior and moral cognition in ethical situations. In particular, moral disengagement is a cognitive tendency (Bandura, 2002; McAlister, 2001; Moore, 2008) that describes individuals temporarily releasing themselves from internal moral constraints through psychological mechanisms such as shifting blame, minimizing the negative consequences of their own behavior, rationalizing behavior (moral justification, sanitizing language, advantageous comparison), and devaluing victims, thereby committing or tolerating immoral behavior (Bandura, 2002). As discussed earlier, workplace ostracism, as a covert stressor, significantly and positively predicts employees' political behavior. Stressful work environments and potentially harmful organizational behaviors are often mediated by moral disengagement, which functions as an individual cognitive mechanism to diminish the ethical attributes of behavior (Bandura, 2002; Moore, 2008). Examples include citizenship pressure and pro-group unethical behavior (Zhang et al., 2023) and workplace ostracism and pro-job unethical behavior (Liu et al., 2025). Therefore, we suppose that the association between workplace ostracism and political behavior may also be mediated by moral disengagement.

Individuals who are experiencing workplace ostracism frequently develop negative emotions such as anger (Tan et al., 2024; Zhu and Zhang, 2021) and anxiety (Samma et al., 2020). Existing research has confirmed that such negative emotional states can serve as critical triggers of moral disengagement (Fida et al., 2015). By using moral disengagement as a cognitive restructuring strategy (Bandura, 1999), individuals can lessen the negative effects of workplace ostracism by justifying their own inappropriate behavior.

Political behavior by employees in organizations can have both positive and negative effects. As a result, this behavior falls under the category of ethical decision-making (Amah, 2022). As a cognitive mechanism, moral disengagement allows individuals to release internal moral constraints and justify or rationalize potentially inappropriate behaviors, such as political behaviors (Bandura, 1999). It enables individuals to participate in such self-serving behaviors without feeling guilty, especially in circumstances involving ego depletion (Cheng et al., 2022). Therefore, we suppose that there is a correlation between moral disengagement and political behavior, which is corroborated by relevant research (Amah, 2022; Zhang et al., 2022).

According to social cognitive theory (Bandura, 1986), employees often use moral disengagement (individual cognition) to safeguard their own interests when they feel that they are being ignored or excluded by others or groups in the workplace (environment). This process encompasses moral justification, displacement of responsibility, and distortion of consequences. This moral cognition weakens the moral constraints on behavior, psychologically removing barriers for employees to engage in self-serving behaviors (even if they may harm the organization or others), ultimately making them more likely to engage in political behavior (individual behavior). Specifically, first, through moral justification, individuals morally justify their political behaviors. Employees who experience workplace ostracism reinterpret their behavioral motives, describing them as self-protective strategies in an unfair environment of being excluded. Such moral justification renders otherwise self-serving or opportunistic political behaviors morally acceptable and reasonable. Second, through displacement of responsibility, employees attribute their behaviors to the external environment (i.e., experienced ostracism) rather than personal responsibility. They perceive that others have violated the implicit norm of fair treatment, hereby attributing their political behaviors to prior unfair exclusion. This mechanism shifts the focus of responsibility away from the self to the external environment, reducing internal moral constraints. Finally, through the distortion of consequences, employees who experience ostracism minimize the potential harm or impropriety of their political behaviors. This distortion reduces the anticipated guilt and moral conflict, allowing employees to engage in political behaviors without self-condemnation. Through these three interrelated processes of moral disengagement, the relationship between workplace ostracism and political behavior is mediated. Such processes enable employees to engage in political behavior without experiencing moral guilt or self-censure.

Hypothesis 2 (H2). Moral disengagement mediates the relationship between workplace ostracism and political behavior.

### 2.3 The moderating role of supervisor-rated job performance

Job performance refers to the behaviors and outcomes employees demonstrate at work that are relevant to organizational goals. This includes both in-role behaviors related to carrying out official job duties and extra-role behaviors that support the organization's effective operation (Williams and Anderson, 1991). This study focuses specifically on supervisor-rated job performance (Smith and Webster, 2017), which captures supervisors' evaluations of their subordinates' work-related behaviors and outcomes. According to the conservation of resources theory, when employees experience workplace ostracism, they face potential or actual losses of social resources (Williams, 2001). Employees may use moral disengagement as a coping strategy in these circumstances to prevent exhausting available resources. When employees receive more positive evaluations, they will receive better rewards, such as higher salaries or better performance ratings (Wang, 2025). These rewards include material resources (e.g., salary) and symbolic resources (e.g., recognition and status), which together constitute the resource reserve that employees can draw upon. Therefore, individuals have a wealth of resources when supervisor-rated job performance is high. These resources can lessen employees' reliance on moral disengagement by mitigating the negative effects of workplace ostracism. This prevents them from coping with stress through strategies that may jeopardize long-term resource accumulation. Conversely, individuals receive fewer positive evaluations and rewards, and their resource reserves are relatively scarce when supervisor-rated job performance is low. Resources might not be enough in these situations to mitigate the negative effects of workplace ostracism. Individuals may depend more and more on moral disengagement to justify their behaviors to prevent further resource depletion (Bandura, 1999; Hobfoll, 1989; Hobfoll et al., 2018).

Hypothesis 3 (H3). Supervisor-rated job performance negatively moderates the relationship between workplace ostracism and moral disengagement.

Drawing from the aforementioned discourse, we suggest that the degree of the association between workplace ostracism and political behavior is moderated by supervisor-rated job performance through moral disengagement. In particular, employees have large reserves of resources such as salary and recognition, when supervisor-rated job performance is high. Employees are less likely to use moral disengagement as a justification for their behaviors, even when they are excluded from the workplace, which eventually lowers the incidence of political behavior. Conversely, employees' resource reserves are relatively scarce when supervisor-rated job performance is low. They not only lack generous salaries but also lack sufficient recognition and status. Moreover, workplace ostracism further depletes these resources. Therefore, they rely on moral disengagement to rationalize and justify their behaviors to prevent further resource loss, which leads to more political behavior.

Hypothesis 4 (H4). Supervisor-rated job performance negatively moderates the mediating role of moral disengagement between workplace ostracism and political behavior.

**Figure 1:**
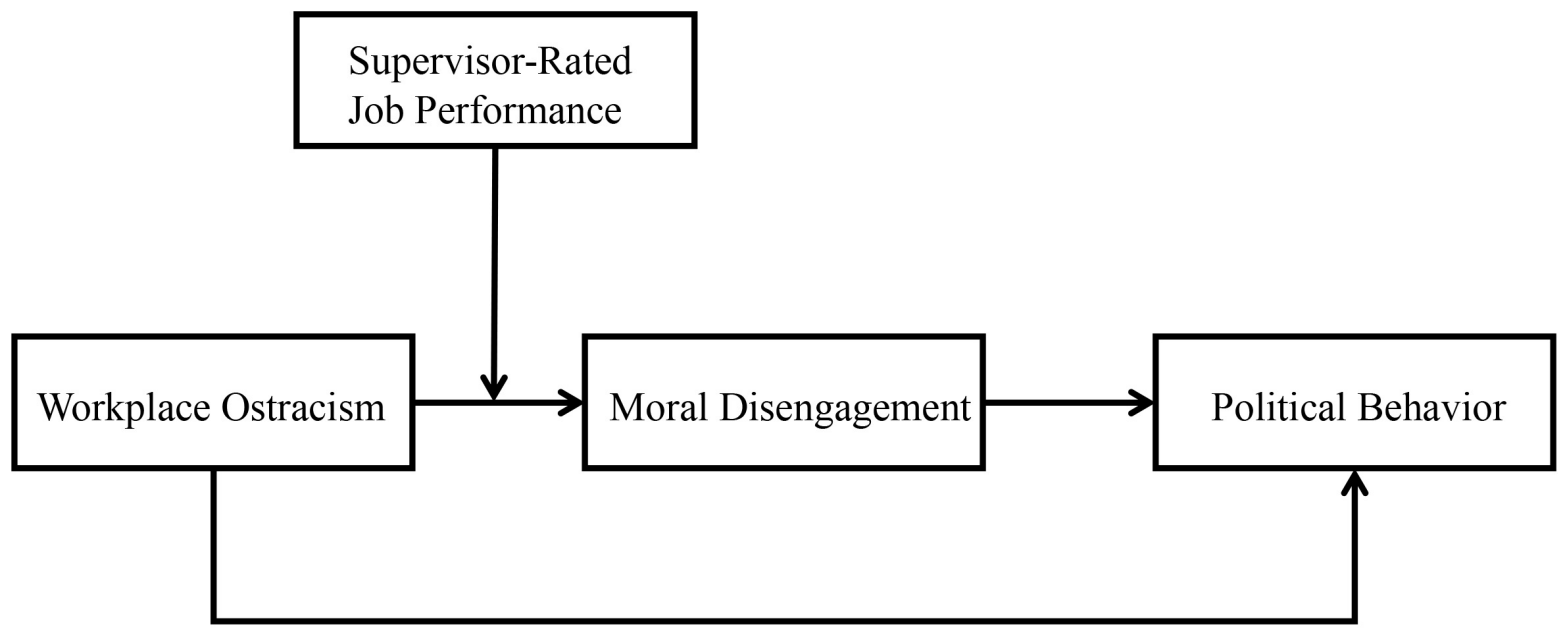
Research model.

The conceptual model of this study is illustrated in Figure 1.

## 3 Materials and methods

### 3.1 Participants and procedures

About three months were spent gathering data for this study, which involved 28 participating organizations. These organizations represented a variety of industries, including manufacturing, information technology, and new energy, and were spread across several Chinese cities, including Shanghai, Chengdu, and Chongqing. Before data collection, the research team discussed the survey design in detail with company contacts and distributed questionnaires after obtaining consent from the company's management. In addition to the accompanying instructions, the team arranged on-site training sessions and provided continuing assistance to answer any questions, ensuring that participants fully understood the content of the questionnaire. In addition to filling out personal information forms, participants had to complete assessments of workplace ostracism, moral disengagement, political behavior, and supervisor-rated job performance. A total of 527 questionnaires were initially gathered in this study. During rigorous data cleaning, 119 questionnaires were excluded for the following specific reasons: 31 questionnaires were excluded due to inconsistent responses, 39 questionnaires were excluded due to early termination (questionnaires left largely unfinished), 40 questionnaires were excluded due to failed attention checks, and 9 questionnaires were excluded due to identical handwriting across multiple questionnaires. After these exclusions, 408 valid questionnaires remained. Among these, 10 questionnaires contained minor missing values, which were subsequently addressed using an appropriate missing data procedure. The final sample of 408 questionnaires was used for formal data analysis, representing an effective response rate of 77.42%. With an average age of 30.13 years, 62% of the 408 participants were women. The average tenure was 6.42 years, 52.9% were married, and 62.5% had a bachelor's degree or above.

Ethical approval for this study was granted by the Ethics Committee of the University of Electronic Science and Technology of China. Prior to data collection, informed consent was obtained from all participants, ensuring that they were fully aware of the study's purpose and their rights.

### 3.2 Measures

This study adopted well-established foreign scales and followed the scale translation and back-translation procedure proposed by Brislin (1986). Except for demographic variables, all variables were measured using a five-point Likert scale, ranging from 1 (strongly disagree) to 5 (strongly agree).

#### 3.2.1 Workplace ostracism

Workplace ostracism was measured using the scale developed by Ferris et al. (2008) and adapted by Thau et al. (2015). A sample item is “I think it is possible that members of my workgroup might treat me as if I am not there.” The Cronbach's alpha for this scale was 0.97. The high alpha value is consistent with prior research. Ferris et al. (2008) reported Cronbach's alpha ranging from 0.89 to 0.96 across four validation samples. However, we still included McDonald's omega as a reliability indicator to support the robustness of the reliability estimates. The results from alpha and omega were highly consistent and nearly identical, supporting the robustness of reliability estimates.

#### 3.2.2 Moral disengagement

Moral disengagement was measured using an 8-item scale developed by Moore et al. (2012). A sample item is “It is okay to spread rumors to defend those you care about.” The Cronbach's alpha for this scale was 0.96. This is a well-established scale commonly showing reliability above 0.90 in the literature. High reliability reflects measurement precision and does not imply redundancy. The omega value for this scale was also 0.96.

#### 3.2.3 Supervisor-rated job performance

The supervisor-rated job performance was measured using a 4-item scale developed by Farh and Cheng (1997). Direct supervisors rated their subordinates, and unique codes were assigned to match each employee-supervisor pair. All responses were kept confidential. A sample item is “This person is one of the best employees in our work unit.” The Cronbach's alpha for this scale was 0.83.

#### 3.2.4 Political behavior

Political behavior was measured using a 6-item scale developed by Treadway et al. (2005). A sample item is “I spend time at work politicking.” The Cronbach's alpha for this scale was 0.92.

#### 3.2.5 Control variables

Control variables included gender, age, education, marital status, and tenure. These variables may be linked to political behavior, according to earlier studies (Amah, 2022; Cheng et al., 2022; Lin et al., 2022; Zhang et al., 2022). Gender was measured as a dichotomous variable, coded as 1 for men and 2 for women. The education level was measured by five categories: high school or vocational school, college, bachelor's degree, master's degree, and doctorate degree, labeled 1–5. The marital status was measured by three categories: unmarried, married, and other, labeled 1–3.

## 4 Results

### 4.1 Confirmatory factor analysis

In this study, MPLUS 7.4 was used to test the discriminant validity through the method of confirmatory factor analysis, and the results are shown in Table 1. Among all the model fitting results, the four-factor benchmark model fit index was the best (χ2/df = 2.776, CFI = 0.950, TLI = 0.945, RMSEA = 0.066, SRMR = 0.038) and better than the competing models, which indicated the high discriminant validity among the four variables.

**Table 1:** Comparison of measurement models.

Models	Factors	χ2	df	χ2/df	RMSEA	CFI	TLI	SRMR
4-factor model	WO, MD, SRJP, PB	746.746	269	2.776	0.066	0.950	0.945	0.038
3-factor model	WO, MD + SRJP, PB	1341.265	272	4.931	0.098	0.889	0.878	0.086
2-factor model	WO + MD + SRJP, PB	2997.820	274	10.941	0.156	0.718	0.691	0.105
1-factor model	WO + MD + SRJP + PB	4235.047	275	15.400	0.188	0.590	0.552	0.139

*N* = 408. WO, workplace ostracism; PB, political behavior; MD, moral disengagement; SRJP, supervisor-rated job performance.

### 4.2 Common method bias testing

This study first used Harman's single-factor test to examine common method bias. The results showed that a total of 14 factors with feature roots >1 were extracted by unrotated exploratory factor analysis. The first factor variance explanation rate was 46.377%, which did not exceed the critical threshold of 50% (Podsakoff and Organ, 1986).

Subsequently, a single-factor confirmatory factor analysis was used, as shown in Table 1. The results showed that the single-factor model had unsatisfactory fitting results (χ2/df = 15.400, RMSEA = 0.188, CFI = 0.590, TLI = 0.552, SRMR = 0.139).

In addition, this study also employed the unmeasured latent method factor approach to test for common method bias. After incorporating the method factor into the model, the fit indices of the model were: χ2/df = 2.447, RMSEA = 0.060, CFI = 0.963, TLI = 0.955, SRMR = 0.030. Compared to the model before control, the improvement in CFI, TLI, RMSEA, and SRMR after adding the method factor was < 0.05, indicating that the fit of the model did not significantly change.

Therefore, it could be concluded that no serious common method bias existed in this study.

### 4.3 Descriptive statistics

The descriptive statistics, including the means, standard deviations, and correlations of study variables, are shown in Table 2. The findings indicated that workplace ostracism was significantly positively correlated with moral disengagement (*r* = 0.680, *p* < 0.01) and political behavior (*r* = 0.472, *p* < 0.01). Moral disengagement was also significantly positively correlated with political behavior (*r* = 0.421, *p* < 0.01). In conclusion, the hypotheses had been preliminarily validated. Notably, workplace ostracism and moral disengagement showed weak or non-significant correlations with demographic control variables. This pattern is consistent with existing meta-analytic evidence. For workplace ostracism, Howard et al. (2020) confirmed that it is largely unrelated to age and tenure, and only weakly associated with gender and education, with negligible effect sizes. For moral disengagement, Gini et al. (2014) demonstrated that it was weakly correlated with gender and age, indicating minimal associations with demographic characteristics. Thus, the non-significant correlations observed in this study are theoretically and empirically reasonable.

**Table 2:** Means, standard deviations, and correlations of variables.

Variables	1	2	3	4	5	6	7	8	9
1. WO	1								
2. MD	0.680^**^	1							
3. PB	0.472^**^	0.421^**^	1						
4. SRJP	0.098^*^	0.026	0.151^**^	1					
5. Gender	−0.033	−0.082	−0.001	0.011	1				
6. Age	0.058	0.038	0.099^*^	0.118^*^	−0.172^**^	1			
7. Education	0.001	−0.013	−0.174^**^	0.057	−0.006	−0.197^**^	1		
8. Marital status	0.059	0.013	0.121^*^	0.127^*^	−0.058	0.571^**^	−0.115^*^	1	
9. tenure	0.037	0.036	0.154^**^	0.120^*^	0.002	0.759^**^	−0.200^**^	0.514^**^	1
M	2.314	1.846	2.972	4.050	1.620	30.130	2.670	1.550	6.423
SD	1.086	1.041	1.094	0.678	0.486	6.605	0.805	0.518	5.845

*N* = 408. ^*^p < 0.05, ^**^p < 0.01. WO, workplace ostracism; PB, political behavior; MD, moral disengagement; SRJP, supervisor-rated job performance.

### 4.4 Hypotheses testing

#### 4.4.1 Main effect testing

This study used Model 4 of the PROCESS macro in SPSS and hierarchical regression analysis to test the direct and indirect effects hypotheses (H1 to H3). Table 3 displays the results of the hierarchical regression. Workplace ostracism had a positive and moderate-to-large effect on political behavior (M1, β = 0.469, *p* < 0.001). Therefore, H1 was validated.

**Table 3:** Regression outcomes.

Variables	PB		MD		
	M1	M2	M3	M4	M5
Gender	−0.003	0.009	−0.068	−0.067	−0.073
Age	−0.117	−0.110	−0.034	−0.032	−0.023
Education	−0.159^***^	−0.156^***^	−0.014	−0.011	−0.008
Marital status	0.057	0.065	−0.041	−0.039	−0.039
Tenure	0.163^*^	0.153^*^	0.055	0.057	0.048
WO	0.469^***^	0.345^***^	0.680^***^	0.683^***^	0.692^***^
MD		0.183^**^			
SRJP				−0.038	−0.049
WO × SRJP					−0.112^**^
*R^2^*	0.270	0.287	0.468	0.469	0.481
Adjusted *R^2^*	0.259	0.275	0.460	0.460	0.471
Δ*R^2^*	0.219	0.018	0.460	0.001	0.012
*F*	24.658^***^	23.034^***^	58.754^***^	39.310^***^	37.047^***^
Δ*F*	120.193^***^	9.978^**^	346.345^***^	1.044	9.358^**^

*N* = 408. ^*^p < 0.05, ^**^p < 0.01, ^***^p < 0.001. WO, workplace ostracism; PB, political behavior; MD, moral disengagement; SRJP, supervisor-rated job performance.

#### 4.4.2 Mediation effect testing

The hierarchical regression analysis results in Table 3 showed that workplace ostracism had a positive and moderate-to-large effect on political behavior (M1, β = 0.469, *p* < 0.001) and a large effect on moral disengagement (M3, β = 0.680, *p* < 0.001). After introducing moral disengagement, moral disengagement had a positive and small-to-moderate effect on political behavior (M2, β = 0.183, *p* < 0.01). Additionally, the effect of workplace ostracism on political behavior (M2, β = 0.345, *p* < 0.001) decreased from 0.469 to 0.345, but the effect remained significant. This indicated that moral disengagement partially mediated the relationship between workplace ostracism and political behavior. Therefore, H2 was validated.

Additionally, Model 4 of the PROCESS macro in SPSS was used to test the mediating effect's significance. The results are shown in Table 4. Through moral disengagement, workplace ostracism had a small-to-moderate indirect effect on political behavior (Indirect effect = 0.124, BootSE = 0.047, 95% BootCI = [0.040, 0.225]). Therefore, H2 was validated once more.

**Table 4:** Bootstrap outcomes.

Path	Effect	BootSE	BootLLCI	BootULCI
Total	0.469	0.042	0.388	0.554
Direct	0.345	0.073	0.198	0.487
Indirect	0.124	0.047	0.040	0.225

#### 4.4.3 Moderation effect testing

**Figure 2:**
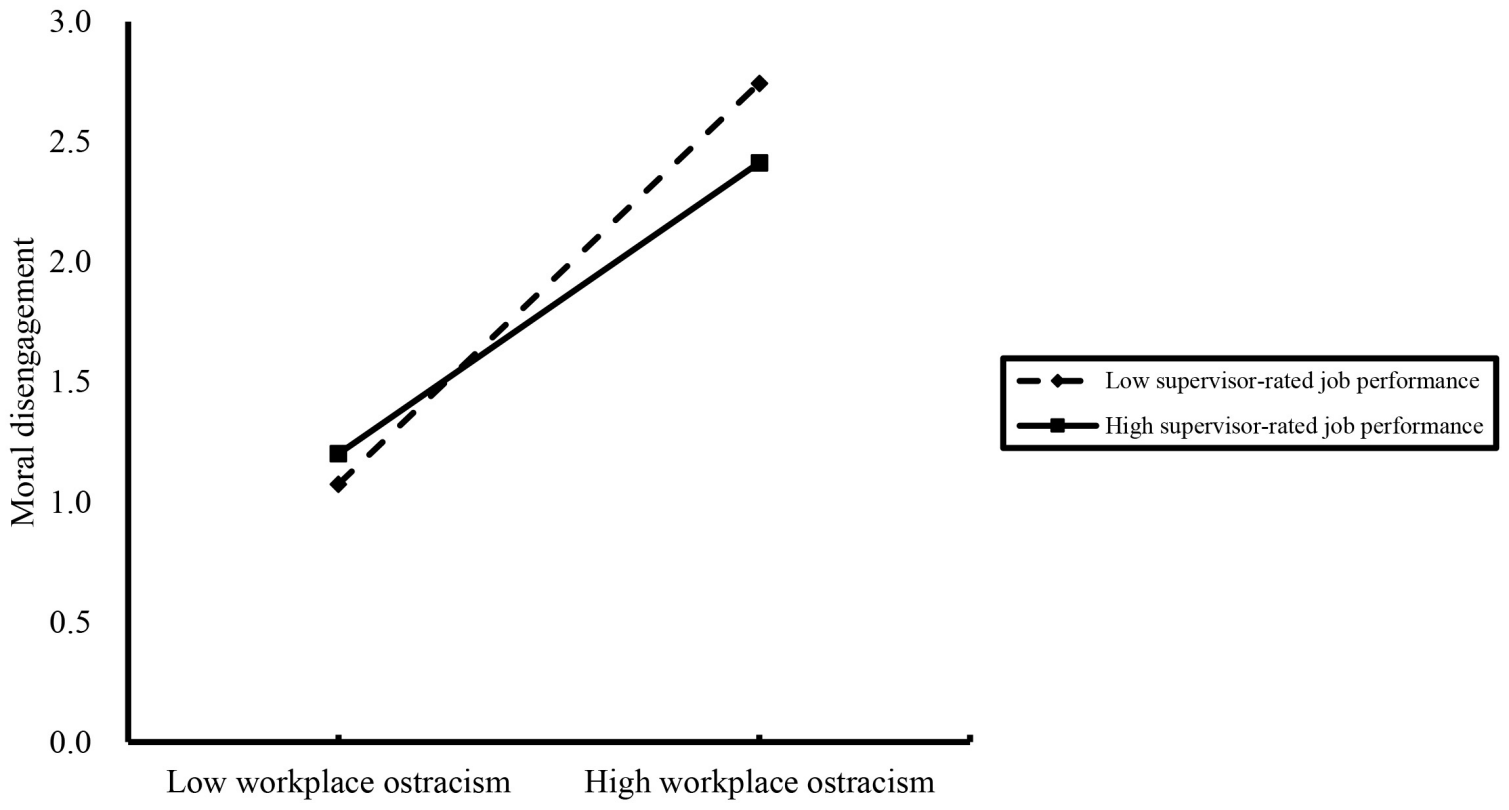
The moderating effect of supervisor-rated job performance on the relationship between workplace ostracism and moral disengagement.

The hierarchical regression analysis results in Table 3 showed that the interaction term between workplace ostracism and supervisor-rated job performance had a negative and small-to-moderate effect on moral disengagement (M5, β = −0.112, *p* < 0.01). This indicated that supervisor-rated job performance moderated the effect of workplace ostracism on moral disengagement. Therefore, H3 was validated. This small-to-moderate interaction effect suggests that supervisor-rated job performance serves as a meaningful but not overpowering buffer in the workplace, meaning it can meaningfully alleviate employees' moral disengagement triggered by ostracism without eliminating this negative experience entirely. To further elucidate the moderating effect, a simple slope analysis was conducted by evaluating the conditional effects at high (M + 1SD) and low (M – 1SD) levels of supervisor-rated job performance. As shown in Figure 2, simple slope analysis revealed that workplace ostracism had a significant and positive effect on moral disengagement for high supervisor-rated job performance (simple slope = 0.558, *p* < 0.001) and low supervisor-rated job performance (simple slope = 0.768, *p* < 0.001). Moreover, compared to the high supervisor-rated job performance group, the low job performance group exhibited a steeper slope, meaning that increased workplace ostracism led to a more significant increase in moral disengagement. Specifically, in a practical workplace context, individuals with low supervisor-rated job performance lack the resources to defend against interpersonal exclusion, making them more likely to loosen their moral standards to cope with unfair treatment. In contrast, individuals with high supervisor-rated job performance enjoy greater status, recognition, and better salary-related rewards in the workplace, which help them maintain moral boundaries even when ostracized.

#### 4.4.4 Moderated mediation effect testing

This study used Model 7 of the PROCESS macro in SPSS to investigate the moderated mediation effect. Overall, supervisor-rated job performance moderated the effect of workplace ostracism on employees' political behavior via moral disengagement (index = −0.298, BootSE = 0.015, 95% BootCI = [−0.061, −0.003]), as shown in Table 5. The positive mediating effect of moral disengagement was significant for low supervisor-rated job performance (Effect = 0.148, BootSE = 0.052, 95% BootCI = [0.050, 0.252]). It was also significant for high supervisor-rated job performance (Effect = 0.107, BootSE = 0.045, 95% BootCI = [0.031, 0.210]). The mediating effect of moral disengagement was much weaker in high supervisor-rated job performance than in low supervisor-rated job performance (contrast = −0.040, BootSE = 0.020, 95% BootCI = [−0.083, −0.004]). Therefore, H4 was validated. Substantively, this means that when employees have low performance ratings, workplace ostracism is more likely to trigger moral disengagement, which in turn leads to political behavior. In contrast, high performance ratings serve as a protective buffer. Employees with strong performance records are less inclined to morally disengage and engage in political behavior when ostracized. From a managerial perspective, these findings suggest that improving employee performance and providing adequate resources (e.g., recognition and support) can mitigate the negative consequences of workplace ostracism.

**Table 5:** The moderated mediation analysis outcomes.

Moderator	Effect	BootSE	BootLLCI	BootULCI
Low supervisor-rated job performance	0.148	0.052	0.050	0.252
High supervisor-rated job performance	0.107	0.045	0.031	0.210
High-low group difference	−0.040	0.020	−0.083	−0.004

## 5 Discussion

This study explores the relationship between workplace ostracism and political behavior, the mediating role of moral disengagement, and the moderating role of supervisor-rated job performance. Drawing on social cognitive theory (Bandura, 1986), our results showed that workplace ostracism positively predicted political behavior, with moral disengagement acting as a mediator. Drawing on conservation of resources theory (Hobfoll, 1989), which accounts for boundary conditions through resource reserves, we further found that the positive effects of workplace ostracism on moral disengagement, as well as the mediating effects between workplace ostracism and political behavior, were negatively reduced by supervisor-rated job performance. Together, these two theories provide a complementary framework, with social cognitive theory explaining the mediating process and conservation of resources theory explaining the boundary condition. All hypotheses proposed were verified.

First, this study showed that workplace ostracism positively predicted political behavior. This result is consistent with previous research (Liu et al., 2025) demonstrating the positive effects of workplace ostracism on neutralizing strategies for self-preservation of employees. Workplace ostracism, as a typical workplace stressor, leads to resource losses for those who are excluded. Political behavior, serving as a crucial strategy for individuals to obtain critical resources (Sun and Chen, 2017; Valle and Perrewe, 2000), enables employees to effectively acquire various instrumental resources. This facilitates resource replenishment and reconstruction in scenarios where resources are compromised. Therefore, employees who are experiencing workplace ostracism may resort to political behavior as a means of self-preservation and resource acquisition.

Further, this study empirically showed that the effect of workplace ostracism on political behavior was mediated by moral disengagement. This indicates that workplace ostracism can not only directly affect political behavior but also increase it by enhancing individual moral disengagement. These findings support the view of social cognitive theory (Bandura, 1986) that external environment (i.e., workplace ostracism), individual cognition (i.e., moral disengagement), and individual behavior (i.e., political behavior) interact with and influence one another. Furthermore, this result coincides with previous empirical studies that reveal the positive link between workplace ostracism and moral disengagement (Liu et al., 2025; Wang et al., 2025) and the positive effect of moral disengagement on political behavior (Zhang et al., 2022).

This study also demonstrated the moderating role of supervisor-rated job performance in both the direct relationship between workplace ostracism and moral disengagement and the indirect effect of workplace ostracism on political behavior via moral disengagement. This moderator remains relatively understudied in the existing literature. These results support the conservation of resources theory's view (Hobfoll et al., 2018), showing that individuals with more abundant resources exhibit lower vulnerability to resource loss and greater capacity for resource gain. At present, the workplace competition is fierce, and workplace ostracism is becoming more and more common, which can easily trigger employees' immoral cognitive and behavioral tendencies (Liu et al., 2025; Qi et al., 2020; Zhang, 2020). The interaction of workplace ostracism and supervisor-rated job performance can reduce their moral disengagement, thereby reducing political behavior. Therefore, supervisor-rated job performance plays an important role in the relationship between workplace ostracism, moral disengagement, and political behavior.

### 5.1 Theoretical implications

First, this study expands the outcome dimensions of workplace ostracism by investigating how employees initiate political behavior when they encounter workplace ostracism. As mentioned earlier, political behavior has two sides. This study found that in negative situations of workplace ostracism, employees tend to adopt dysfunctional political behaviors as coping strategies. Although such behaviors may lead to individuals experiencing a higher degree of emotional labor (Treadway et al., 2005) or result in interpersonal deviance (Barlett et al., 2016), for employees who have suffered workplace exclusion, when there are no other effective ways to deal with the situation, such behaviors still have certain practical significance in striving for their own rights and improving their circumstances. This provides a new perspective on the rationality of political behavior among excluded employees, creating new opportunities to investigate the causes and real-world consequences of political behavior in organizations.

Second, this study reveals the underlying mechanism that links workplace ostracism and political behavior, emphasizing the mediating role of moral disengagement in this relationship. This role is consistent with the theoretical framework of social cognitive theory (Bandura, 1986). Workplace ostracism is a common environmental factor that may trigger moral disengagement as a cognitive tendency, leading to political behavior. The study advances knowledge of the underlying logic through which workplace ostracism affects political behavior by emphasizing this mediating role of moral disengagement.

Finally, this study reveals the boundary conditions of workplace ostracism on political behavior. The association between workplace ostracism and employees' performance has been thoroughly studied in the past, but these studies mainly considered employees' performance as an outcome variable (Imran et al., 2023; Kuo and Wu, 2022; Wang and Lai, 2023). However, we suggest that supervisor-rated job performance is likely to have a major moderating effect on the relationship between workplace ostracism, moral disengagement, and political behavior, given the special features of the workplace environment and the crucial role that performance rating plays in organizational management. Furthermore, superiors' ratings of job performance can more accurately gauge the moderating effect of workplace ostracism on the process of moral disengagement affecting employees' political behavior because they are the employees' direct supervisors and main points of contact in the workplace.

### 5.2 Practical implications

First, this study reveals that workplace ostracism has a positive effect on employees' political behavior. This finding provides a potential behavioral reference for employees who are experiencing workplace ostracism. When facing workplace ostracism, political behavior may help them advocate for their rights and improve their situation. However, it is important to note that political behavior has two sides. Although it may have functional value in certain contexts, the dysfunctional political behavior focused on in this study, due to its self-serving nature, may be harmful to both individuals and organizations in the long run (Treadway et al., 2005). Therefore, political behavior can only be used as a last resort for employees to cope with workplace ostracism, that is, it should only be considered when other more constructive coping strategies (e.g., seeking supervisor support, building positive peer relationships) are unavailable or have proven ineffective. In addition to actively communicating with employees and attempting to reduce workplace ostracism, managers must cultivate an equitable, just, peaceful, and encouraging work environment. Organizations should also set up strong channels for employee feedback so that every employee has a way to voice their concerns and resolve grievances.

Second, this study reveals that employees who are experiencing workplace ostracism may behave politically through moral disengagement. Therefore, one of the most important ways to stop such political behavior is to lower the level of moral disengagement among employees. Organizations should first create explicit codes of behavior, bolster oversight and transparency procedures, and give employees ethical standards in order to successfully reduce moral disengagement. Employee ethical development should be a top priority for organizations. Organizations can strengthen employees' internal moral compass by improving their capacity for ethical scrutiny and judgment through ongoing and successful ethics education, awareness campaigns, and training. This strategy successfully lowers moral disengagement brought on by exclusion from the workplace, which in turn lowers political behavior.

Finally, the results of this study show that the effect of workplace ostracism on moral disengagement and the indirect effects resulting from moral disengagement are weaker the higher the supervisor-rated job performance. Therefore, in order to prevent rating imbalances brought on by personal biases, emotional factors, and other influences, organizations should set up fair and impartial performance evaluation systems. Additionally, organizations shouldn't focus only on performance metrics since this could make underperforming employees feel like a waste of resources, which would make them more likely to engage in moral disengagement and political behavior. Rather, organizations should show humanistic concern by giving employees the resources and support they need to perform better. More encouragement and guidance should be given to underperforming employees.

### 5.3 Limitations and future directions

Because of the cross-sectional design used in this study, it is impossible to precisely determine the dynamic and causal relationships between workplace ostracism and political behavior. Reverse causality (e.g., political behavior predicting workplace ostracism) is possible and should be examined in future longitudinal research. However, it is important to clarify that reverse causality addresses a different research question than the one this study focuses on. This study is grounded in social cognitive theory, which conceptualizes workplace ostracism as a situational stressor that triggers cognitive and behavioral responses. The primary interest is in how employees respond to experienced workplace ostracism, specifically whether moral disengagement mediates the link from workplace ostracism to political behavior. While we acknowledge that political behavior may also lead to workplace ostracism, this alternative direction does not undermine our theoretical contribution, as it pertains to the antecedents rather than the consequences of ostracism. Nevertheless, future research using longitudinal designs is needed to test both directions and clarify temporal dynamics.

Individual traits like Machiavellian personality (Dogan, 1997; Liu, 2010) and proactive personality (Zhao et al., 2013) may also play significant roles in the process by which workplace ostracism affects political behavior, in addition to the moderating role of supervisor-rated job performance previously discussed. Regarding contextual factors, perceived organizational support is conceptualized as an environmental work resource that can help individuals replenish the resources depleted due to stress (Sarwar et al., 2020). Given that workplace ostracism can be regarded as a stressor, perceived organizational support may also play a buffering role in the relationship between workplace ostracism and political behavior. Furthermore, there are other ways to deal with workplace ostracism besides the particular coping strategy for political behavior we have looked at. Future research should therefore look into more factors that might affect the relationship between workplace ostracism and political behavior, as well as more efficient coping strategies.

The study was carried out in a Chinese context. The results may have limitations due to the effect of cultural differences on individual attitudes and behaviors. Therefore, to compare the relationship between workplace ostracism and political behavior across cultural contexts, future research could expand its scope by using cross-national samples. Additionally, although the sample was centered on the Chinese context, relationship mechanisms specific to the Chinese local context, like supervisor-subordinate guanxi, were not fully incorporated into the model construction and variable selection (Ma et al., 2023). Therefore, in order to more thoroughly examine the effects of workplace ostracism on political behavior, future research could go deeper into the causal pathways of these relationship mechanisms.

Finally, although participants were recruited from 28 organizations, indicating a hierarchical structure with employees nested within organizations, this study used traditional regression-based models (PROCESS) that treat observations as independent. Future research could adopt multilevel modeling to account for the nested data structure and obtain more robust estimates.

## Data Availability

The raw data supporting the conclusions of this article will be made available by the authors, without undue reservation.
